# Sodium arsenite and hyperthermia modulate cisplatin-DNA damage responses and enhance platinum accumulation in murine metastatic ovarian cancer xenograft after hyperthermic intraperitoneal chemotherapy (HIPEC)

**DOI:** 10.1186/1757-2215-4-9

**Published:** 2011-06-22

**Authors:** Clarisse S Muenyi, Vanessa A States, Joshua H Masters, Teresa W Fan, C William Helm, J Christopher States

**Affiliations:** 1 Department of Pharmacology & Toxicology, University of Louisville, Louisville, KY 40292, USA; 2 Department of Chemistry, University of Louisville, Louisville, KY 40292, USA; 3 Center for Regulatory and Environmental Analytical Metabolomics, University of Louisville, Louisville, KY 40292, USA; 4 Center for Genetics & Molecular Medicine, University of Louisville, Louisville, KY 40292, USA; 5 Center for Environmental Genomics & Integrative Biology, University of Louisville, Louisville, KY 40292, USA; 6 James Graham Brown Cancer Center, University of Louisville, Louisville, KY 40292, USA; 7 Department of Obstetrics and Gynecology, Division of Gynecologic Oncology, St. Louis University School of Medicine, St Louis, MO 63117, USA

**Keywords:** cisplatin, sodium arsenite, hyperthermia, HIPEC, metastatic human ovarian cancer, p53, XPA, XPC, MSH2, platinum accumulation

## Abstract

**Background:**

Epithelial ovarian cancer (EOC) is the leading cause of gynecologic cancer death in the USA. Recurrence rates are high after front-line therapy and most patients eventually die from platinum (Pt) - resistant disease. Cisplatin resistance is associated with increased nucleotide excision repair (NER), decreased mismatch repair (MMR) and decreased platinum uptake. The objective of this study is to investigate how a novel combination of sodium arsenite (NaAsO_2_) and hyperthermia (43°C) affect mechanisms of cisplatin resistance in ovarian cancer.

**Methods:**

We established a murine model of metastatic EOC by intraperitoneal injection of A2780/CP70 human ovarian cancer cells into nude mice. We developed a murine hyperthermic intraperitoneal chemotherapy model to treat the mice. Mice with peritoneal metastasis were perfused for 1 h with 3 mg/kg cisplatin ± 26 mg/kg NaAsO_2 _at 37 or 43°C. Tumors and tissues were collected at 0 and 24 h after treatment.

**Results:**

Western blot analysis of p53 and key NER proteins (ERCC1, XPC and XPA) and MMR protein (MSH2) suggested that cisplatin induced p53, XPC and XPA and suppressed MSH2 consistent with resistant phenotype. Hyperthermia suppressed cisplatin-induced XPC and prevented the induction of XPA by cisplatin, but it had no effect on Pt uptake or retention in tumors. NaAsO_2 _prevented XPC induction by cisplatin; it maintained higher levels of MSH2 in tumors and enhanced initial accumulation of Pt in tumors. Combined NaAsO_2 _and hyperthermia decreased cisplatin-induced XPC 24 h after perfusion, maintained higher levels of MSH2 in tumors and significantly increased initial accumulation of Pt in tumors. ERCC1 levels were generally low except for NaAsO_2 _co-treatment with cisplatin. Systemic Pt and arsenic accumulation for all treatment conditions were in the order: kidney > liver = spleen > heart > brain and liver > kidney = spleen > heart > brain respectively. Metal levels generally decreased in systemic tissues within 24 h after treatment.

**Conclusion:**

NaAsO_2 _and/or hyperthermia have the potential to sensitize tumors to cisplatin by inhibiting NER, maintaining functional MMR and enhancing tumor platinum uptake.

## Background

Epithelial ovarian cancer (EOC) is the leading cause of gynecological cancer death in the U.S. Approximately 22,000 women are diagnosed annually and 15,000 die from the disease [1]. Most women are diagnosed only after peritoneal dissemination has occurred. The standard treatment for patients with EOC is cytoreductive surgery (CRS) followed by intravenous Pt-taxane chemotherapy [2]. Even though initially effective, relapse from residual disease and/or drug resistant cancer reduces the 5-year survival rate to about 20% [3]. Despite research efforts to improve on Pt-based chemotherapy, or to develop new drugs against EOC, most patients still die from metastatic disease. Since metastatic EOC is usually confined in the peritoneal cavity, it makes theoretical sense to deliver chemotherapy intraperitoneally rather than intravenously since higher levels of drug can be delivered to the disease site by that route [4,5]. In response to three large randomized clinical trials showing benefit to incorporating intraperitoneal (IP) delivery in EOC, the National Cancer Institute issued a clinical announcement recommending that patients with small volume disease at the end of frontline surgery be offered the chance of receiving IP chemotherapy [6]. Adding hyperthermia to chemotherapy agents delivered intraperitoneally (HIPEC) theoretically could improve outcome [7-9].

Cisplatin is a DNA damaging chemotherapeutic used to treat solid tumors including EOC. However, resistance to cisplatin limits clinical success. Mechanisms of cisplatin resistance are multi-factorial and include reduced cellular drug accumulation, enhanced drug metabolism by glutathionylation and export by multidrug resistance proteins, enhanced DNA damage tolerance and DNA repair [10]. Since Pt-containing chemotherapy drugs remain the major weapon against EOC, improving their efficacy could have a great impact on mortality. The combination of hyperthermia with cisplatin has been reported for the treatment of EOC [11]. Hyperthermia is tumoricidal alone [12] and has been shown to enhance cisplatin inhibition of peritoneal tumor growth by increasing tumor Pt accumulation [13]. Arsenic trioxide (As_2_O_3_), an FDA approved drug for the treatment of all-trans-retinoic acid-resistant acute promyelocytic leukemia [14] has the potential to sensitize tumors to cisplatin [15,16]. Combination chemotherapy studies demonstrate that arsenic sensitizes cancer cells to hyperthermia, radiation, cisplatin, adriamycin, doxorubicin, and etoposide [16-19]. *In vitro *studies demonstrate that trivalent arsenic (As^3+ ^administered as arsenic trioxide [As_2_O_3_, Trisenox^®^] or sodium arsenite [NaAsO_2_]) induces apoptosis in multiple types of cancer cells including cervical, melanoma, gastric, colon, pancreatic, lung, prostate and ovarian cancer cell lines [20-23]. *In vivo *studies also show that arsenic inhibits the growth of orthotopic metastatic prostate cancer and peritoneal metastatic ovarian cancer [24,25]. The mechanism of arsenic-induced cell death *in vitro *is suggested to include formation of oxidative DNA damage [26], activation of the Fas pathway [27], inhibition of DNA repair [28,29], and causation of mitotic arrest and induction of apoptosis in the mitotic cells [20,21].

As^3+ ^has biological effects similar to those of both cisplatin and hyperthermia. Like cisplatin it is detoxified by glutathionylation and exported by multidrug resistant family transport pumps [30,31], suggesting a potential for competition for the detoxification pathway if arsenic and cisplatin are used in combination. This competition might enhance cisplatin accumulation in cells. Like hyperthermia, As^3+ ^induces stress response proteins and causes mitotic catastrophe [21]. These actions make arsenic a potentially effective agent to augment hyperthermia enhancement of cisplatin-induced cell death.

The goal of this study is to determine how sodium arsenite and hyperthermia modulate mechanisms of cisplatin resistance *in vivo*. We developed murine models of HIPEC treatment and metastatic human EOC to investigate if NaAsO_2 _and hyperthermia alter the expression of DNA repair proteins and tumor platinum levels. We show that NaAsO_2 _and hyperthermia either as single agents or in combination reverse key DNA repair protein responses to cisplatin responsible for cisplatin resistance and also enhanced tumor Pt uptake suggesting decreased Pt detoxification.

## Methods

### Chemicals

Cisplatin and sodium arsenite were purchased from Sigma-Aldrich (St. Louis, MO). Stock solutions (cisplatin 1 mg/mL in 1X PBS and NaAsO_2 _13 mg/mL in water) were prepared freshly on the day of treatment and filter sterilized (0.22 μm) prior to use.

### Cells and cell culture

Cisplatin-resistant (A2780/CP70) human ovarian cancer cells were the kind gift of Dr. Eddie Reed. Cells were maintained in RPMI 1640 medium containing 10% fetal bovine serum, 100 μg/mL penicillin/streptomycin, 2 mM L-glutamine and 0.2 units/mL insulin. Cells were cultured in an atmosphere of 95% humidity and 5% CO_2 _at 37°C. Cells were passaged twice weekly and replated at a density of 1 × 10^6 ^cells/150 mm dish.

### Animals

Female NCr athymic nude mice (7 - 9 weeks old), were purchased from Taconic (Cambridge City, IN). Animals were kept in a temperature-controlled room on a 12 h light-dark schedule. The animals were maintained in cages with paper filter covers under controlled atmospheric conditions. Cages, covers, bedding, food, and water were changed and sterilized weekly. Animals were fed autoclaved animal chow diet and water. All procedures were performed under sterile conditions. This experiment was approved by the Institutional Animal Care and Use Committee of the University of Louisville in an AALAC approved facility in accordance with all regulatory guidelines.

### Establishment of intraperitoneal metastatic ovarian tumors in mice

**Figure 1:**
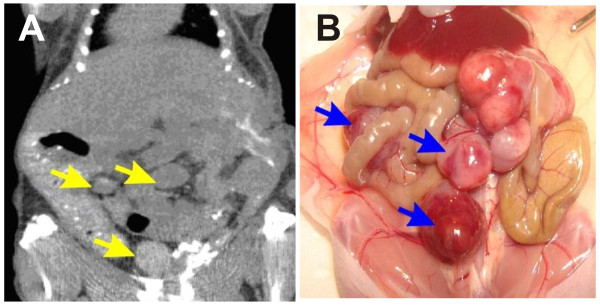
**Mouse with multiple small intraperitoneal tumors**. **A. **MicroCT scan of tumors in live mouse. **B. **Direct visualization of tumors at necropsy of mouse. Three tumors are denoted by arrow in panels **A **and **B**.

A2780/CP70 cell suspension (1 × 10^6 ^cells in 500 μL of serum-free RPMI 1640 media) was injected into the peritoneum of anesthetized mice using an 18-gauge needle. The needle was flushed with 500 μL physiological saline. The abdomen of injected animals was massaged to ensure even distribution of cells. By 3 - 4 weeks after injection, the mice had developed multiple small disseminated IP tumors (1 - 7 mm) (Figure 1). Tumors were monitored by microCT scanning in the Brown Cancer Center Small Animal Imaging Facility.

### Intraperitoneal chemotherapy

**Figure 2:**
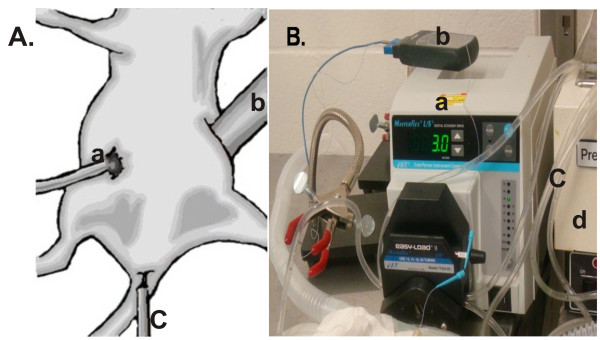
**Murine hyperthermic intraperitoneal chemotherapy model**. **A. **Drawing of tumor bearing mouse undergoing HIPEC. Depicted are inlet (a) and outlet (b) ports and anal temperature probe (c) to monitor internal temperature of mouse during perfusion. **B. **Photograph showing perfusion pump (a), temperature monitor (b), flow tubes (c) and heating bath (d). Mice were perfused for 1 h at the rate of 3 mL/min with cisplatin (3 mg/kg) ± NaAsO_2 _(26 mg/kg) at 37 or 43°C.

Tumor-bearing mice were anesthetized with 3% isoflurane in an inhalation chamber and maintained on 1% isoflurane during surgery. Incisions (~0.5 cm) were made on both sides of the lower abdominal wall allowing entry into the peritoneal cavity (Figure 2). Inflow and outflow tubes were inserted into the peritoneal cavity and secured with skin sutures. The tubes were connected to a bag containing 100 mL normal saline with added cisplatin (3 mg/kg body weight (BW)) ± sodium arsenite (26 mg/kg BW) and cefazolin (0.01 mg/mL). (The dose of cisplatin used for this study was determined from human dose of cisplatin (100 mg/m^2^) administered intravenously to a 70 kg (body surface area = 1.87 m^2^) [32] cancer patient and sodium arsenite dose was calculated from a single daily dose of Trisenox (0.15 mg/kg/day) administered intravenously to a 70 kg acute promyelocytic leukemia patient. The underlying assumption in the calculations is that the drugs are mixed in 2 L saline solution for HIPEC therapy). The saline bag was submerged in a water bath to maintain the perfusate temperature at either 37 or 43°C. Perfusion was performed at a rate of 3 mL/min for 60 min using a Masterflex pump (Cole-Palmer Instrument Co, Cat # 07524-50). The inflow and outflow temperatures were monitored by thermocouple probes with temperature maintained within 1°C. The core temperature of the animals was monitored using an anal temperature probe and maintained using a heating pad and heat lamp. After 60 min perfusion, most of the perfusate in the peritoneum was sucked out using sterile cotton balls with a light abdominal massage. Wounds were sutured closed and animals were injected intraperitoneally with 1 mL physiological saline containing 0.01 mg ketoprofen for pain. Mice were kept in warm cages (single mouse/cage) and monitored for recovery and discomfort. Immediately (0 h) and 24 h after perfusion, mice were euthanized and tumors, kidneys, liver, spleen, heart and brain were dissected and snap frozen in liquid nitrogen and stored at -80°C until use.

### Western blot analysis

Tumors of ~ 3-5 mm in diameter were homogenized in protein lysis solution (1 M Tris-HCl pH 7.4, 0.5 M EDTA, 10% sodium dodecyl sulfate, 180 μg/mL phenylmethylsulphonylfluoride) using a tissue grinder. After removal of debris by centrifugation (45 min, 14,000 x g), total protein concentration in supernatant was determined by bicinchoninic acid (BCA) method according to manufacturer's instructions (Pierce, Rockford, IL, micro-well plate protocol) [33]. Fifteen μg protein samples were resolved by SDS-polyacrylamide gel electrophoresis and electro-transferred to nitrocellulose membranes. Membranes were probed with antibodies to XPA (Neomarkers, MS-650-P1, dilution 1:1000), XPC (Novus, # ab6264, dilution 1:10,000), GAPDH (Sigma, # A 5441, dilution 1:10,000), p53 (DO-1, Cell Signaling Technology, # 9284, dilution 1:1000), MSH2 (Santa Cruz, # SC-494, dilution 1:1000), and ERCC1 (Santa Cruz, # SC-10785, dilution 1:1000). Secondary antibodies (rabbit anti-mouse IgG, # 81-6120 or goat anti-rabbit, # 81-6120, dilutions 1:2500) conjugated to horseradish peroxidase (Zymed Laboratories, Inc. South San Francisco, CA) were bound to primary antibodies and protein bands detected using enhanced chemiluminescence (ECL) substrate (Pierce, Rockford, IL). GAPDH was used as the loading control. Films were scanned with a Molecular Dynamics Personal Densitometer SI (Molecular Dynamics, Sunnyvale, CA) and analyzed with ImageQuaNT software (Molecular Dynamics) to determine band density.

### ICP-MS analysis

Samples of tumor homogenates were lyphophilized using Heto vacuum centrifuge (ATR, Laurel, MD) and 350 μL concentrated nitric acid was added to each sample. Wet weight of brain, heart, spleen, liver and kidney was recorded and concentrated nitric acid (350 - 500 μL) was added to samples. Samples were predigested overnight, and then 100 μL of each dissolved sample was transferred into 10 mL acid washed microwavable digestion tubes (triplicate for each sample). The samples were microwave-digested at 150°C for 10 min using an automated focused beam microwave digestion system (Explorer™, CEM, Matthews, NC, USA). After digestion, 1.9 mL of 18 Mohm H_2_O containing 10 ppb internal standard (SPEX CertiPrep, Metuchen, NJ) was added into every sample to give final 5% nitric acid and ICP-MS analyses was performed using Thermo X Series II ICP-MS (Thermo Fisher Scientific, Waltham, MA) at the University of Louisville Center for Regulatory and Environmental Analytical Metabolomics facility. Concentrated nitric acid was processed similarly as blank. Platinum standard (SPEX CertiPrep, Metuchen, NJ) was used to generate a standard curve. Platinum and arsenic levels in tumors and tissues were expressed as ng metal/mg protein and ng metal/mg wet weight respectively. Results are presented as the means of three ICP-MS determinations for each data point ± SD from 3 individual mice.

### Immunocytochemistry

Cells (1 × 10^5 ^) were plated on poly-D-lysine coated coverslips (BD Biosciences) in a 24-well plate and allowed to acclimate for 24 h. Cells were then treated with 40 μM cisplatin for 1 h. After treatment, cells were washed twice with PBS and incubated in drug-free media for 24 h. Cells were fixed in ice-cold acetone for 10 min at room temperature and washed twice with ice cold PBS and samples incubated for 10 min with PBS containing 0.25% Triton X-100 (PBST). Cells were then washed with PBS three times for 5 min and incubated in 3% hydrogen peroxide for 30 min to quench endogenous peroxidase. Cells were washed three times with PBS and incubated in 1% BSA in PBST for 30 min to block unspecific binding of the antibodies. Cells were incubated overnight at 4°C in primary antibodies (1:200 dilution in PBST containing 1% BSA). The primary antibodies used were XPA (Neomarkers, MS-650-P1), XPC (H-300, SantaCruz Biotechnology, # sc-30156), p53 (DO-1, Cell Signaling Technology, # 9284), MSH2 (Santa Cruz, # SC-494) and ERCC1 (Santa Cruz, # SC-10785). After incubation, the primary antibody solution was decanted and cells were washed three times with PBS for 5 min each wash. Cells were incubated with secondary antibodies (rabbit anti-mouse IgG, # 81-6120 or goat anti-rabbit, # 81-6120, dilution 1:200 in PBST containing 1% BSA) conjugated to horseradish peroxidase (Zymed Laboratories, Inc. South San Francisco, CA) for 1 h at room temperature. Secondary antibody solution was decanted and cells were washed three times with PBS for 5 min. Cells were stained with 3,3'-diaminobenzidine (DAB) substrate solution by incubating cells in 200 μL premixed DAB solution (mix 30 μL (one drop) of the DAB liquid chromogen solution to 2 mL of the DAB liquid buffer solution (Sigma, # D 3939)) for 10 min. DAB solution was removed and cells rinsed briefly with PBS. Cells were counterstained with 20% Wright Giemsa solution for 1 min. Coverslips were mounted on microscope slides using a drop of permount mounting medium. Slides were viewed under a Nikon Eclipse E600 Microscope (Fryer Company Inc, Scientific Instruments, Cincinnati, OH 45240) and pictures taken using MetaMorph software (Universal Imaging Corporation). DAB-positive cells were counted per 1000 cells using MetaMorph software.

### Statistical analysis

Statistical analyses were performed using wilcoxon rank sum test with significance set as p < 0.05, n ≥ 3.

## Results

### Murine intraperitoneal chemotherapy model

Multiple disseminated tumors were established in the peritoneal cavity of nude mice as described in Materials and Methods. Mice were scanned using microCT scan to determine the location and estimate the size of tumors (Figure 1A). This was confirmed upon necropsy (Figure 1B). Tumor bearing mice were treated by peritoneal lavage for 1 h with cisplatin ± sodium arsenite at 37°C (normothermia) or 43°C (hyperthermia) (Figures 2A and 2B) as described in Materials and Methods. During treatment, the required inflow temperature was reached within 2-5 min after the start of perfusion. Inflow, outflow and rectal temperatures were recorded every 15 min and remained stable within 1°C throughout the 60 min perfusion (Table 1).

**Table 1:** Inflow, outflow and body temperatures of mouse during intraperitoneal perfusion

Inflow Temperature	Outflow Temperature	Body Temperature
37.4 ±1.1°C	36.4 ± 0.8°C	35.5 ± 1.0°C
43.0 ± 0.7°C	39.7 ± 0.6°C	36.3 ± 2.1°C

Mice were perfused for 1 h with cisplatin (CP/37; CP/43) or cisplatin + NaAsO2 (CPA/37; CPA/43) at 37 or 43°C respectively. Inflow, outflow and body temperatures were recorded every 15 min. Data are presented as means ± SD of readings taken from five mice.

### Platinum and arsenic accumulation and retention in metastatic tumors

**Figure 3:**
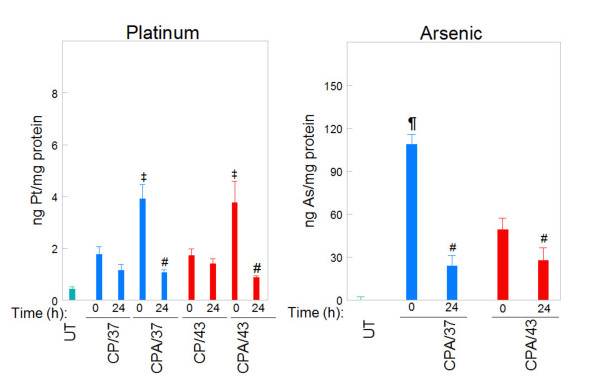
**Inductively Coupled Plasma Mass Spectrometry (ICP-MS) determination of platinum and arsenic in tumors**. Mice were perfused for 1 h with cisplatin (CP/37; CP/43) or cisplatin + NaAsO_2 _(CPA/37; CPA/43) at 37 or 43°C respectively. Tumors from untreated (UT) and treated mice were harvested at 0 and 24 h after treatment. Tumors were homogenized and samples of the homogenate were analyzed for protein concentration by BCA or digested in nitric acid for ICP-MS analysis for platinum and arsenic. Data are presented as means ± SEM of ≥3 tumors each from different mice. Statistical analysis was performed using wilcoxon rank sum test. P < 0.05, N ≥ 3: # = lower than 0 h partner, ‡ = higher than CP/37 at 0 h and CP/43 at 0 h, ¶ = higher than CPA/43°C at 0 h.

We determined Pt and arsenic accumulation in tumors immediately (0 h) and 24 h after perfusion using ICP-MS. Pt and arsenic accumulated in tumors during treatment (0 h) and generally decreased after treatment (24 h), compared with the untreated control (Figure 3). Co-treatment with NaAsO_2 _and cisplatin at 37°C (CPA/37) or 43°C (CPA/43) caused significantly more Pt to accumulate in tumors. By 24 h after perfusion, tumor Pt levels for CPA/37 and CPA/43 treatment conditions decreased to levels similar to CP/37. Hyperthermia did not increase tumor Pt levels nor alter Pt retention in tumors 24 h after treatment. More arsenic initially accumulated in tumors when co-treated with cisplatin and NaAsO_2 _at 37°C (CPA/37) than with hyperthermia treatment (CPA/43). Arsenic decreased to similar levels at 24 h.

### Effect of cisplatin, arsenic and hyperthermia on DNA repair protein expression

**Figure 4:**
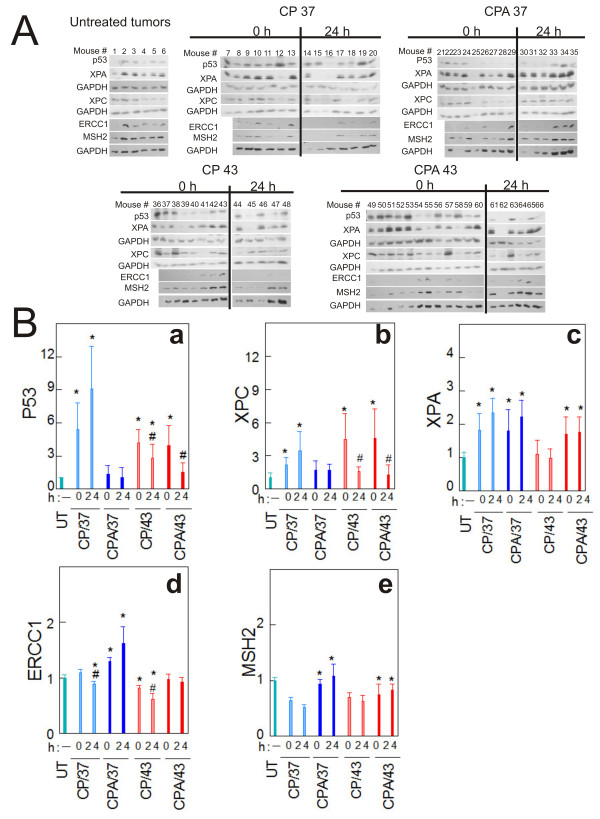
**DNA repair protein expression in tumors**. **A. **Western blot determination of p53, XPC, XPA, ERCC1 and MSH2 in tumors. GAPDH is loading control. **B**. Densitometry analyses of (**a**) p53, (**b**) XPC, (**c**) XPA, (**d**) ERCC1 and (**e**) MSH2 normalized to GAPDH loading control and untreated tumors. Mice were perfused for 1 h with cisplatin (CP/37; CP/43) or cisplatin plus NaAsO_2 _(CPA/37; CPA/43) at 37 or 43°C respectively. Tumors from untreated (UT) mice and treated mice were harvested 0 and 24 h after treatment. Protein extracts were prepared from the tumors and 20 μg loaded per lane for SDS-PAGE. Data are presented as means ± SD of ≥5 tumors each from different mice. Statistical analysis was performed using wilcoxon rank sum test. P < 0.05, N ≥ 5. # = compared to 0 h partner, * = compared to UT.

Cisplatin causes bulky DNA damage that is repaired mostly by the nucleotide excision repair system (NER). Cellular response to cisplatin-DNA damage involves the induction of DNA repair proteins to initiate DNA repair [10]. We determined if NaAsO_2 _and hyperthermia modulated the expression of XPC, a platinum-DNA damage recognition protein in global genome repair (GGR) [34] subpathway of NER, and of ERCC1 and XPA, downstream NER proteins that have been implicated in cisplatin resistance [35]. We also determined the expression of p53, which is involved in the activation of the GGR pathway by transcriptionally activating XPC [36]. In addition to NER, decreased mismatch repair (MMR) has been implicated in cisplatin resistance [37,38]. Thus, we also investigated the expression of MSH2, an important MMR DNA damage recognition protein. Western blot analysis of p53, XPC, XPA, ERCC1 and MSH2 revealed mouse-to-mouse and tumor-to-tumor variabilities (Figure 4A). Some tumors failed to express the protein of interest while others either expressed high, moderate or very low levels of the proteins. We determined band intensities for the expressed proteins by scanning the films using a Molecular Dynamics Personal Densitometer SI (Molecular Dynamics, Sunnyvale, CA) and analyzing bands of interest using ImageQuaNT software (Molecular Dynamics). Each protein value was normalized to its respective GAPDH (loading control) value. Data were further normalized to untreated control (Figure 4B). Tumors that failed to express the protein of interest were not considered in the densitometry analyses. P53 (Figure 4B, panel a) and XPC (Figure 4B, panel b) were significantly induced during treatment (0 h) by cisplatin at 37°C (CP/37) or 43°C (CP/43) and cisplatin plus arsenite at 43°C (CPA/43). P53 significantly decreased at 24 h after treatment with CPA/43 (Figure 4B, panel a). XPC decreased at 24 h after perfusion with both CP/43 and CPA/43 treatments (Figure 4B, panel b). P53 (Figure 4B, panel a) and XPC (Figure 4B, panel b) did not significantly increase during (0 h) and after (24 h) peritoneal lavage with NaAsO_2 _and cisplatin co-treatment at 37°C (CPA/37). XPA (Figure 4B, panel c) was significantly induced during (0 h) and 24 h after perfusion with CP/37, CPA/37 and CPA/43 but not with CP/43. ERCC1 remained generally low for all treatment conditions except with CPA/37 (Figure 4B, panel d). The suppression of MSH2 by CP/37 and CP/43 treatments was not seen in tumors co-treated with arsenite (CPA/37, CPA/43) (Figure 4B, panel e).

### Expression of P53, XPA and MSH2 in ovarian cancer cells

**Figure 5:**
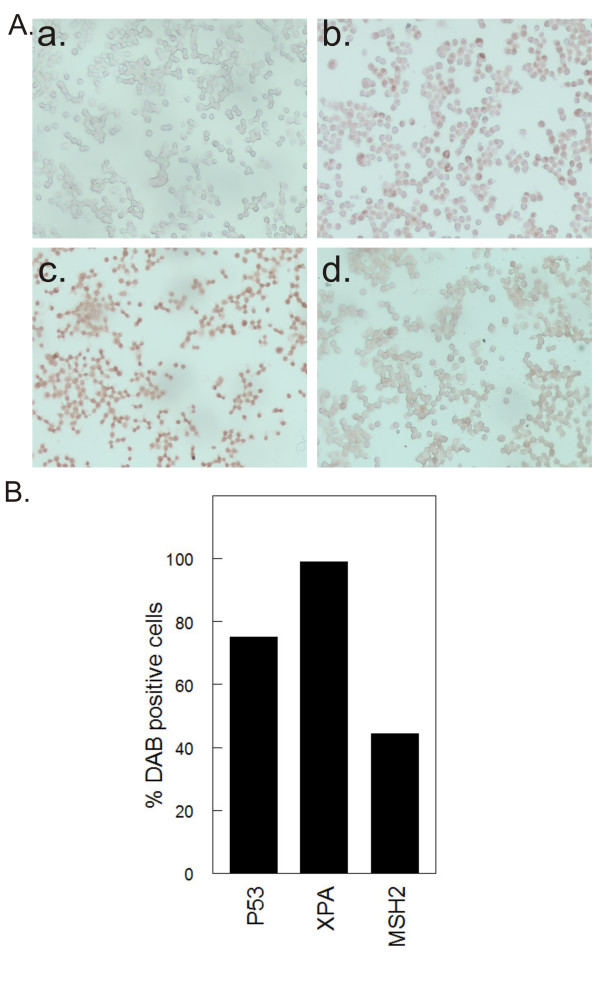
**Immunocytochemical determination of p53, XPA and MSH2 expression in ovarian cancer cells**. **A. **A2780/CP70 cells were treated for 1 h with 40 μM cisplatin. Cells were washed and incubated in drug-free media for 24 h and immunohistochemistry was performed. Representative pictures of cells at 20x magnification for secondary antibody only control (a), p53 (b), XPA (c) and MSH2 (d). **B. **Plot of 3,3'-diaminobenzidine (DAB)-positive cells. Data are single biological experiment performed in duplicate slides. Four different fields were counted per coverslip.

Western blot determination of P53, XPC, XPA, ERCC1 and MSH2 in metastatic tumors revealed that some tumors failed to express p53 (6%), XPC (3%), XPA (8%), ERCC1 (40%) and MSH2 (9%). Failure to express these proteins could be an inherent feature of the cells that were used to establish the tumors or due to mutations and alteration of genes during tumor development that could result in lack of protein expression. We therefore performed immunocytochemical studies using A2780/CP70 cells to determine expression of P53, XPA and MSH2 in these cells (Figure 5A). Immunocytochemistry data revealed that 25% of cells do not express p53 as evident by lack of 3,3'-diaminobenzidine (DAB) brown staining and ~3% and 60% of cells did not stain positive for XPA and MSH2 respectively (Figure 5B). Full-length western blots for XPC and ERCC1 had several non-specific bands in addition to the band of interest (data not shown) making it impossible to perform immunocytochemistry with specificity for these proteins.

### Platinum and arsenic biodistribution in somatic tissues

**Figure 6:**
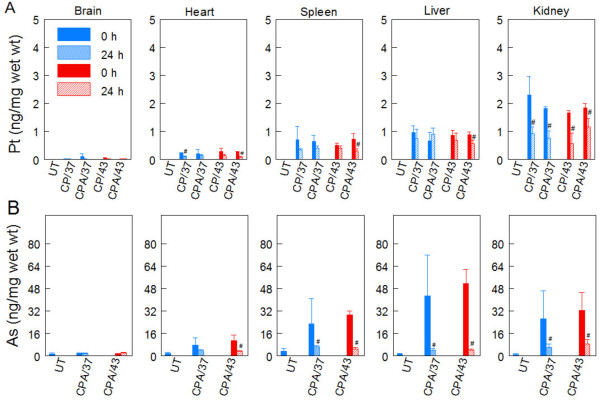
**Platinum and arsenic accumulation in somatic tissues**. Mice were perfused for 1 h with cisplatin (CP/37; CP/43) or cisplatin + NaAsO_2 _(CPA/37; CPA/43) at 37 or 43°C respectively. Tissues from untreated (UT) and treated mice were harvested at 0 and 24 h after treatment. Tissue samples were weighed and digested in nitric acid for ICP-MS analysis for platinum (**A**) and arsenic (**B**). Data are presented as means ± SD of triplicate samples each from different mice. Statistical analysis was performed using wilcoxon rank sum test. P < 0.05, N = 3. # = compared to 0 h partner.

The clinical use of anticancer chemotherapeutic agents is limited by adverse toxicities. For cisplatin, these include toxicity to the kidney, peripheral nerves, liver, heart, bone marrow and brain [39,40]. Clinical use of arsenic is known to cause liver, kidney and neurological damage, cardiovascular and gastro-intestinal toxicity, anemia and leucopenia [41-43]. Therefore, we determined cisplatin and arsenic accumulation in mouse tissues including kidney, liver, heart, spleen and brain (Figure 6A and 6B). Samples were prepared as described in Methods. During perfusion, platinum accumulated in all tissues examined regardless of the treatment condition, in the order: kidney > liver = spleen > heart > brain. At 24 h after perfusion, significant decrease of platinum was observed in the kidney for all treatment conditions. The combination treatment (CPA/43) favored the removal of platinum from the liver, spleen and heart at 24 h after perfusion. Arsenic also significantly accumulated in all the tissues examined, in the order: liver > kidney = spleen > heart > brain and it significantly decreased in all tissues by 24 h after perfusion.

## Discussion

Although the platinum analogues (cisplatin and carboplatin) are at the forefront of combination treatment for EOC, acquired or inherent resistance limits clinical success. In the current study, we used metastatic EOC xenograft in nude mice to investigate how NaAsO_2 _and hyperthermia modulate response to cisplatin *in vivo*. We focused on three key mechanisms of cisplatin resistance: enhanced NER, diminished MMR and decreased Pt accumulation. Our data suggest that cisplatin induces resistant phenotype in metastatic tumors by inducing XPC and XPA and suppressing MSH2. Sodium arsenite alone or combined with hyperthermia inhibits mechanisms of cisplatin resistance by suppressing XPC induction, maintaining higher levels of MSH2 and increasing tumor uptake of cisplatin.

Decreased Pt accumulation is an important mechanism of cisplatin resistance. Hyperthermia has been reported to increase both cellular and DNA Pt levels*in vitro*. However, *in vivo *data remains controversial. Los et al used rats bearing metastatic colon cancer to show that hyperthermia suppressed tumor growth by increasing platinum accumulation in tumors [13]. Zeamari et al used a similar colon cancer xenograft model in rats and reported that hyperthermia did not increase tumor Pt levels [44]. Similar to Zeamari, we observed that hyperthermia does not increase Pt accumulation in tumors. The observed discrepancies with Los et al could be due to differences in how HIPEC was performed. Los et al injected hyperthermic cisplatin intraperitoneally; whereas we and Zeamari et al performed peritoneal lavage similar to what is done clinically. Unlike hyperthermia, we observed that NaAsO_2 _at 37 or 43°C increased initial tumor Pt levels. Since arsenic and cisplatin are detoxified by glutathionylation and export by the multidrug resistant family proteins, potential competition for the detoxification/export pathways might have resulted in more Pt accumulating in the tumors when cisplatin is co-administered with sodium arsenite.

Cisplatin is a DNA damaging agent and p53 is implicated in platinum-DNA damage response [36]. P53 is frequently mutated in ovarian cancer [45]. The p53 phenotype of A2780/CP70 cells remains controversial. Some studies have demonstrated that A2780/CP70 cells have non-functional p53 [46,47], while other studies have shown that these cells have wild type p53 [48,49]. Our data indicate that A2780/CP70 cell population is heterogeneous: ~75% of cells express wild type p53 and ~25% are p53 null (Figure 5). In addition, 6% of the tumors derived from A2780/CP70 are p53 null (Figure 4A). Our *in vitro *data also demonstrate the induction of p53 target genes p21CIP1/WAF1, XPC and DDB2 in A2780/CP70 cells (data not shown), which strongly suggests that a large fraction of these cells have wild type p53. The observed heterogeneity might have resulted from mutations and alterations that occur during serial propagation of cells in culture leading to cell line drift [50]. The observed heterogeneity may impact response to chemotherapy and result in treatment failures because p53 wild type and null cells will respond differently to chemotherapy especially DNA damaging agents such as cisplatin. This heterogeneity explains why targeting master regulators such as p53 or AKT in cancer cells has not been successful [51,52]. Therefore, combination chemotherapy such as cisplatin, sodium arsenite and hyperthermia with different mechanisms of action might be more beneficial than using a single drug to target a single protein or pathway.

Cisplatin predominantly forms intrastrand DNA crosslinks that are repaired by the nucleotide excision repair (NER) system. There are two subpathways of NER; transcription coupled repair (TCR) which removes damage from actively transcribing DNA and global genome repair (GGR) which removes lesions from the entire genome [53]. These two pathways differ only in the proteins that are involved in damage recognition. In TCR, CSA and CSB along with RNA pol II recognize damage, whereas in GGR, XPC and DDB2 are important for lesion recognition. XPC is actively involved in the recognition and initiation of cisplatin-DNA damage repair in GGR [34,54]. Arsenic has been shown to inhibit NER by inhibiting XPC expression [29]. In the current study, we observed that P53 and XPC were induced by cisplatin. However, NaAsO_2 _alone or in combination with hyperthermia prevented the induction of p53 and XPC by cisplatin (Figure 4B, panels a and b). Since p53 is known to transcriptionally induce XPC [36], our data suggest that NaAsO_2 _± hyperthermia might be inhibiting p53, which in turn might be suppressing XPC induction. Suppression of XPC will potentially sensitize tumors to cisplatin. Our *in vitro *data suggest that inhibition of XPC using siRNA sensitizes ovarian cancer cells to cisplatin (data not shown). Therefore, the suppression of XPC could potentially sensitize tumors to cisplatin in a similar fashion. Following DNA damage recognition, downstream DNA repair proteins (XPA, RPA, TFIIH complex, ERCC1/XPF and XPG) are recruited to the DNA damage recognition complexes in both TCR and GGR to remove the damage in a common pathway. Over-expression of XPA and ERCC1 mRNA has been associated with cisplatin resistance in ovarian cancer [35]. In the current study, cisplatin induced XPA (Figure 4B, panel c) that was suppressed by hyperthermia co-treatment (Figure 4 panel c). Suppression of XPA might decrease repair of cisplatin-DNA damage. ERCC1 was modestly induced (<1.5 fold) by NaAsO_2 _co-treatment with cisplatin at 37°C (CPA37) (Figure 4B, panel d).

In addition to the NER pathway, the mismatch repair (MMR) system has been implicated in cisplatin resistance [37]. In an effort to repair Pt-DNA damage by the MMR system, a futile MMR occurs leading to cell death [53,55]. Ovarian cancer cells over-expressing MMR proteins are sensitive to cisplatin [55-57]. We report for the first time that tumors treated with cisplatin at 37°C (CP37) significantly suppressed MSH2 consistent with resistance. The observed suppression of MSH2 by cisplatin was reversed in tumors co-treated with NaAsO_2 _at 37 or 43°C (CPA/37 and CPA/43 respectively) Thus, NaAsO_2 _at 37 or 43°C has the potential to sensitize tumors to cisplatin by maintaining functional MMR.

Cisplatin causes serious and dose-limiting side effects including kidney damage, peripheral sensory neuropathy, cardiovascular toxicity, myelosuppression and anemia which occur as a result of diffusion of chemotherapy from the peritoneal to systemic compartment. In addition, arsenic also causes adverse side effects including cardiovascular toxicity, kidney damage, myelosuppression and anemia, liver damage and peripheral sensory neuropathy. Understanding the biodistribution of these drugs during peritoneal perfusion of chemotherapy is important in order to predict the occurrence of these adverse side effects and determine the risk:benefit balance in performing intraperitoneal perfusion with cisplatin and arsenic. For this reason, we determined platinum and arsenic accumulation in the brain, heart, liver, kidney and spleen during (0 h) and 24 h after perfusion. We observed that platinum and arsenic accumulated to similar extent in these tissues regardless of the treatment condition. The greatest accumulation of Pt was observed in the kidney, the site of Pt elimination. Likewise, greatest level of arsenic was observed in the liver, the organ for arsenic metabolism and detoxification. Even though we did not observe any toxicity with the short-term survival study, accumulation of arsenic and Pt in assayed organs suggests that potential adverse side effects such as encephalopathy, cardiotoxicity, liver damage, renal damage and myelosuppression/anemia respectively may occur during long-term survival studies.

## Conclusions

NaAsO_2 _alone or combined with hyperthermia is most likely to enhance cisplatin efficacy because of its abilities to impair NER by inhibiting induction of p53 and XPC and to activate MMR by maintaining high levels of MSH2 and enhancing platinum accumulation in tumors. NaAsO_2 _and hyperthermia might not produce added systemic toxicity to cisplatin chemotherapy; on the contrary, the combined treatment might help in the clearance of Pt from tissues. Long-term survival studies are required to determine the efficacy of this new combination chemotherapy. The murine HIPEC model may serve as a useful tool to study in vivo mechanisms of platinum resistance and explore ways to sensitize tumors to platinum chemotherapy.

## Abbreviations

CP: (cisplatin); CP/37: (cisplatin at 37°C) or CP/43 (cisplatin at 43°C); CPA: (cisplatin plus sodium arsenite); CPA37: (cisplatin plus sodium arsenite at 37°C) or CPA/43 (cisplatin plus sodium arsenite at 43°C); ERCC1: (excision repair cross-complementing 1); GGR: (global genome repair); HIPEC: (hyperthermic intraperitoneal chemotherapy); ICP-MS: (inductively coupled plasma mass spectrometry); NaAsO_2_: (sodium arsenite); MSH2: (human mutS homolog 2); NER: (nucleotide excision repair); Pt: (platinum); TCR: (transcription coupled repair); XPA: (xeroderma pigmentosum group A); XPC: (xeroderma pigmentosum group C).

## Competing interests

Dr Helm has previously received speaking honoraria from ThermaSolutions and grant support from ThermaSolutions and Sanofi-Aventis for clinical research into Hyperthermic Intraperitoneal Chemotherapy for the treatment of ovarian carcinoma.

All other authors declare that they have no competing interests

## Authors' contributions

CSM established metastatic tumor model, performed HIPEC and tissue collection, ICP-MS analysis, western blot analysis, immunohistochemical studies and drafted the manuscript. VAS established metastatic tumor model, performed HIPEC and tissue collection, took and drew pictures for figures 1 and 2. JHM established metastatic tumor model, developed murine HIPEC model in collaboration with CWH, performed HIPEC and tissue collection. TWF provided intellectual input with ICP-MS analysis. CWH developed murine HIPEC model, established metastatic tumor model and participated in study design, coordination, data analysis and manuscript editing. JCS developed murine HIPEC model, established metastatic tumor model and participated in study design, coordination, data analysis and manuscript editing. All authors read and approved the final manuscript.

## References

[B1] Jemal A Siegel R Ward E Hao Y Xu J Thun MJ Cancer statistics, 2009 CA Cancer J Clin 2009 59 225 249 10.3322/caac.20006 19474385

[B2] Armstrong DK Bundy B Wenzel L Huang HQ Baergen R Lele S Copeland LJ Walker JL Burger RA Intraperitoneal cisplatin and paclitaxel in ovarian cancer N Engl J Med 2006 354 34 43 10.1056/NEJMoa052985 16394300

[B3] Ozols RF Treatment goals in ovarian cancer Int J Gynecol Cancer 2005 15 Suppl 1 3 11 15839952 10.1111/j.1525-1438.2005.15351.x

[B4] Markman M Intraperitoneal chemotherapy in the management of malignant disease Expert Rev Anticancer Ther 2001 1 142 148 10.1586/14737140.1.1.142 12113122

[B5] van d V van d V Zoetmulder FA van Goethem AR van T O ten Bokkel Huinink WW Beijnen JH Bartelink H Begg AC Intraperitoneal cisplatin with regional hyperthermia in advanced ovarian cancer: pharmacokinetics and cisplatin-DNA adduct formation in patients and ovarian cancer cell lines Eur J Cancer 1998 34 148 154 10.1016/S0959-8049(97)00370-5 9624250

[B6] Trimble EL Christian MC National Cancer Institute-United States strategy regarding intraperitoneal chemotherapy for ovarian cancer Int J Gynecol Cancer 2008 18 Suppl 1 26 28 18336395 10.1111/j.1525-1438.2007.01100.x

[B7] Helm CW The role of hyperthermic intraperitoneal chemotherapy (HIPEC) in ovarian cancer Oncologist 2009 14 683 694 10.1634/theoncologist.2008-0275 19608639

[B8] Yang XJ Li Y Yonemura Y Cytoreductive surgery plus hyperthermic intraperitoneal chemotherapy to treat gastric cancer with ascites and/or peritoneal carcinomatosis: Results from a Chinese center J Surg Oncol 2010 101 457 464 10.1002/jso.21519 20401915

[B9] Dovern E de Hingh IH Verwaal VJ van Driel WJ Nienhuijs SW Hyperthermic intraperitoneal chemotherapy added to the treatment of ovarian cancer. A review of achieved results and complications Eur J Gynaecol Oncol 2010 31 256 261 21077465

[B10] Cepeda V Fuertes MA Castilla J Alonso C Quevedo C Perez JM Biochemical mechanisms of cisplatin cytotoxicity Anticancer Agents Med Chem 2007 7 3 18 10.2174/187152007779314044 17266502

[B11] Helm CW Bristow RE Kusamura S Baratti D Deraco M Hyperthermic intraperitoneal chemotherapy with and without cytoreductive surgery for epithelial ovarian cancer J Surg Oncol 2008 98 283 290 10.1002/jso.21083 18726895

[B12] Giovanella BC Stehlin JS Jr Morgan AC Selective lethal effect of supranormal temperatures on human neoplastic cells Cancer Res 1976 36 3944 3950 975042

[B13] Los G van Vugt MJ Pinedo HM Response of peritoneal solid tumours after intraperitoneal chemohyperthermia treatment with cisplatin or carboplatin Br J Cancer 1994 69 235 241 10.1038/bjc.1994.45 8297720 PMC1968708

[B14] Cohen MH Hirschfeld S Flamm HS Ibrahim A Johnson JR O'Leary JJ White RM Williams GA Pazdur R Drug approval summaries: arsenic trioxide, tamoxifen citrate, anastrazole, paclitaxel, bexarotene Oncologist 2001 6 4 11 11161223 10.1634/theoncologist.6-1-4

[B15] Helm CW States JC Enhancing the efficacy of cisplatin in ovarian cancer treatment - could arsenic have a role J Ovarian Res 2009 2 2 10.1186/1757-2215-2-2 19144189 PMC2636805

[B16] Wang W Qin SK Chen BA Chen HY Experimental study on antitumor effect of arsenic trioxide in combination with cisplatin or doxorubicin on hepatocellular carcinoma World J Gastroenterol 2001 7 702 705 11819858 10.3748/wjg.v7.i5.702 PMC4695578

[B17] Chun YJ Park IC Park MJ Woo SH Hong SI Chung HY Kim TH Lee YS Rhee CH Lee SJ Enhancement of radiation response in human cervical cancer cells in vitro and in vivo by arsenic trioxide (As2O3) FEBS Lett 2002 519 195 200 10.1016/S0014-5793(02)02765-5 12023044

[B18] Griffin RJ Monzen H Williams BW Park H Lee SH Song CW Arsenic trioxide induces selective tumour vascular damage via oxidative stress and increases thermosensitivity of tumours Int J Hyperthermia 2003 19 575 589 10.1080/0265673031000124316 14756449

[B19] Uslu R Sanli UA Sezgin C Karabulut B Terzioglu E Omay SB Goker E Arsenic trioxide-mediated cytotoxicity and apoptosis in prostate and ovarian carcinoma cell lines Clin Cancer Res 2000 6 4957 4964 11156257

[B20] McNeely SC Belshoff AC Taylor BF Fan TW McCabe MJ Jr Pinhas AR States JC Sensitivity to sodium arsenite in human melanoma cells depends upon susceptibility to arsenite-induced mitotic arrest Toxicol Appl Pharmacol 2008 229 252 261 10.1016/j.taap.2008.01.020 18328521 PMC2474465

[B21] Taylor BF McNeely SC Miller HL States JC Arsenite-induced mitotic death involves stress response and is independent of tubulin polymerization Toxicol Appl Pharmacol 2008 230 235 246 10.1016/j.taap.2008.02.030 18485433 PMC2504415

[B22] Cui X Kobayashi Y Akashi M Okayasu R Metabolism and the paradoxical effects of arsenic: carcinogenesis and anticancer Curr Med Chem 2008 15 2293 2304 10.2174/092986708785747526 18781951

[B23] Murgo AJ Clinical trials of arsenic trioxide in hematologic and solid tumors: overview of the National Cancer Institute Cooperative Research and Development Studies Oncologist 2001 6 Suppl 2 22 28 11331437 10.1634/theoncologist.6-suppl_2-22

[B24] Maeda H Hori S Nishitoh H Ichijo H Ogawa O Kakehi Y Kakizuka A Tumor growth inhibition by arsenic trioxide (As2O3) in the orthotopic metastasis model of androgen-independent prostate cancer Cancer Res 2001 61 5432 5440 11454688

[B25] Zhang J Wang B Arsenic trioxide (As(2)O(3)) inhibits peritoneal invasion of ovarian carcinoma cells in vitro and in vivo Gynecol Oncol 2006 103 199 206 10.1016/j.ygyno.2006.02.037 16624393

[B26] Nakagawa Y Akao Y Morikawa H Hirata I Katsu K Naoe T Ohishi N Yagi K Arsenic trioxide-induced apoptosis through oxidative stress in cells of colon cancer cell lines Life Sci 2002 70 2253 2269 10.1016/S0024-3205(01)01545-4 12005185

[B27] Kong B Huang S Wang W Ma D Qu X Jiang J Yang X Zhang Y Wang B Cui B Yang Q Arsenic trioxide induces apoptosis in cisplatin-sensitive and -resistant ovarian cancer cell lines Int J Gynecol Cancer 2005 15 872 877 10.1111/j.1525-1438.2005.00251.x 16174238

[B28] Hartwig A Groblinghoff UD Beyersmann D Natarajan AT Filon R Mullenders LH Interaction of arsenic(III) with nucleotide excision repair in UV-irradiated human fibroblasts Carcinogenesis 1997 18 399 405 10.1093/carcin/18.2.399 9054635

[B29] Nollen M Ebert F Moser J Mullenders LH Hartwig A Schwerdtle T Impact of arsenic on nucleotide excision repair: XPC function, protein level, and gene expression Mol Nutr Food Res 2009 10.1002/mnfr.200800480 19382146

[B30] Leslie EM Haimeur A Waalkes MP Arsenic transport by the human multidrug resistance protein 1 (MRP1/ABCC1). Evidence that a tri-glutathione conjugate is required J Biol Chem 2004 279 32700 32708 10.1074/jbc.M404912200 15161912

[B31] Stewart DJ Mechanisms of resistance to cisplatin and carboplatin Crit Rev Oncol Hematol 2007 63 12 31 10.1016/j.critrevonc.2007.02.001 17336087

[B32] Reagan-Shaw S Nihal M Ahmad N Dose translation from animal to human studies revisited FASEB J 2008 22 659 661 17942826 10.1096/fj.07-9574LSF

[B33] Smith PK Krohn RI Hermanson GT Mallia AK Gartner FH Provenzano MD Fujimoto EK Goeke NM Olson BJ Klenk DC Measurement of protein using bicinchoninic acid Anal Biochem 1985 150 76 85 10.1016/0003-2697(85)90442-7 3843705

[B34] Neher TM Rechkunova NI Lavrik OI Turchi JJ Photo-cross-linking of XPC-Rad23B to cisplatin-damaged DNA reveals contacts with both strands of the DNA duplex and spans the DNA adduct Biochemistry 2010 49 669 678 10.1021/bi901575h 20028083 PMC2811759

[B35] Dabholkar M Vionnet J Bostick-Bruton F Yu JJ Reed E Messenger RNA levels of XPAC and ERCC1 in ovarian cancer tissue correlate with response to platinum-based chemotherapy J Clin Invest 1994 94 703 708 10.1172/JCI117388 8040325 PMC296149

[B36] Ford JM Regulation of DNA damage recognition and nucleotide excision repair: another role for p53 Mutat Res 2005 577 195 202 15927209 10.1016/j.mrfmmm.2005.04.005

[B37] Fink D Zheng H Nebel S Norris PS Aebi S Lin TP Nehmé A Christen RD Haas M MacLeod CL Howell SB In vitro and in vivo resistance to cisplatin in cells that have lost DNA mismatch repair Cancer Res 1997 57 1841 1845 9157971

[B38] Jensen KC Mariappan MR Putcha GV Husain A Chun N Ford JM Schrijver I Longacre TA Microsatellite instability and mismatch repair protein defects in ovarian epithelial neoplasms in patients 50 years of age and younger Am J Surg Pathol 2008 32 1029 1037 10.1097/PAS.0b013e31816380c4 18469706

[B39] Hartmann JT Lipp HP Toxicity of platinum compounds Expert Opin Pharmacother 2003 4 889 901 10.1517/14656566.4.6.889 12783586

[B40] Gesson-Paute A Ferron G Thomas F de Lara EC Chatelut E Querleu D Pharmacokinetics of oxaliplatin during open versus laparoscopically assisted heated intraoperative intraperitoneal chemotherapy (HIPEC): an experimental study Ann Surg Oncol 2008 15 339 344 10.1245/s10434-007-9571-9 17943387

[B41] Senkus E Jassem J Cardiovascular effects of systemic cancer treatment Cancer Treat Rev 2010 10.1016/j.ctrv.2010.11.001 21126826

[B42] Emadi A Gore SD Arsenic trioxide - An old drug rediscovered Blood Rev 2010 24 191 199 10.1016/j.blre.2010.04.001 20471733 PMC2918685

[B43] Au WY Kwong YL Arsenic trioxide: safety issues and their management Acta Pharmacol Sin 2008 29 296 304 10.1111/j.1745-7254.2008.00771.x 18298894

[B44] Zeamari S Floot B van d V Stewart FA Pharmacokinetics and pharmacodynamics of cisplatin after intraoperative hyperthermic intraperitoneal chemoperfusion (HIPEC) Anticancer Res 2003 23 1643 1648 12820435

[B45] Berchuck A Kohler MF Marks JR Wiseman R Boyd J Bast RC Jr The p53 tumor suppressor gene frequently is altered in gynecologic cancers Am J Obstet Gynecol 1994 170 246 252 8296829 10.1016/s0002-9378(94)70414-7

[B46] Jones NA Turner J McIlwrath AJ Brown R Dive C Cisplatin- and paclitaxel-induced apoptosis of ovarian carcinoma cells and the relationship between bax and bak up-regulation and the functional status of p53 Mol Pharmacol 1998 53 819 826 9584207

[B47] Lu X Errington J Curtin NJ Lunec J Newell DR The impact of p53 status on cellular sensitivity to antifolate drugs Clin Cancer Res 2001 7 2114 2123 11448931

[B48] Brown R Clugston C Burns P Edlin A Vasey P Vojtesek B Kaye SB Increased accumulation of p53 protein in cisplatin-resistant ovarian cell lines Int J Cancer 1993 55 678 684 10.1002/ijc.2910550428 8406999

[B49] Yazlovitskaya EM DeHaan RD Persons DL Prolonged wild-type p53 protein accumulation and cisplatin resistance Biochem Biophys Res Commun 2001 283 732 737 10.1006/bbrc.2001.4849 11350044

[B50] Hughes P Marshall D Reid Y Parkes H Gelber C The costs of using unauthenticated, over-passaged cell lines: how much more data do we need? Biotechniques 2007 43 575 577-2 10.2144/000112598 18072586

[B51] Zeimet AG Marth C Why did p53 gene therapy fail in ovarian cancer? Lancet Oncol 2003 4 415 422 10.1016/S1470-2045(03)01139-2 12850192

[B52] Engelman JA Targeting PI3K signalling in cancer: opportunities, challenges and limitations Nat Rev Cancer 2009 9 550 562 10.1038/nrc2664 19629070

[B53] Martin LP Hamilton TC Schilder RJ Platinum resistance: the role of DNA repair pathways Clin Cancer Res 2008 14 1291 1295 10.1158/1078-0432.CCR-07-2238 18316546

[B54] Earley JN Turchi J Interrogation of nucleotide excision repair capacity: Impact on platinum-based cancer therapy Antioxid Redox Signal 2010 10.1089/ars.2010.3369 PMC3096502 20812782

[B55] Topping RP Wilkinson JC Scarpinato KD Mismatch repair protein deficiency compromises cisplatin-induced apoptotic signaling J Biol Chem 2009 284 14029 14039 10.1074/jbc.M809303200 19286655 PMC2682851

[B56] Ding X Mohd AB Huang Z Baba T Bernardini MQ Lyerly HK Berchuck A Murphy SK Buermeyer AB Devi GR MLH1 expression sensitises ovarian cancer cells to cell death mediated by XIAP inhibition Br J Cancer 2009 101 269 277 10.1038/sj.bjc.6605180 19603033 PMC2720211

[B57] Pani E Stojic L El-Shemerly M Jiricny J Ferrari S Mismatch repair status and the response of human cells to cisplatin Cell Cycle 2007 6 1796 1802 10.4161/cc.6.14.4472 17622800

